# Dietary Fats Substitution and Blood Pressure Levels: A Longitudinal Study in Mexican Adults

**DOI:** 10.3390/nu17132096

**Published:** 2025-06-24

**Authors:** Paola Villaverde, Berenice Rivera-Paredez, Rafael Velázquez-Cruz, Anna D. Argoty-Pantoja, Jorge Salmerón

**Affiliations:** 1 Research Center in Policies, Population and Health, School of Medicine, National Autonomous University of Mexico (UNAM), Mexico City 04510, Mexico; paovila@gmail.com (P.V.); bereriveraparedez7@gmail.com (B.R.-P.); 2 Genomics of Bone Metabolism Laboratory, National Institute of Genomic Medicine (INMEGEN), Mexico City 14610, Mexico; rvelazquez@inmegen.gob.mx; 3 Department of Epidemiology, University of Groningen, University Medical Center Groningen, 9713 GZ Groningen, The Netherlands

**Keywords:** hypertension, Mexican population, adults, macronutrients, energy intake, carbohydrates, fats, proteins, substitution models

## Abstract

**Background:** Dietary patterns impact blood pressure (BP) levels, but the potential impact of replacing specific types of fats with proteins or carbohydrates, in isocaloric models, on BP remains unclear. **Objective:** This study evaluates the longitudinal association between the substitution of different types of fats with proteins or carbohydrates and changes in BP in a Mexican population. **Methods:** We analyzed data from 1448 adults (mean age at baseline: 45 years; 73.3% women) from the Health Workers Cohort Study, followed over 13 years. Trained personnel measured systolic (SBP) and diastolic (DBP) blood pressure following standard procedures and techniques at baseline and follow-up. Macronutrient intake was assessed with a validated semi-quantitative food frequency questionnaire. Generalized Estimating Equations (GEE) for hypertension and fixed-effects linear regression for BP were conducted using isocaloric substitution models. Each estimate reflects the effect of a 3% energy substitution of specific fats for carbohydrates or proteins. **Results:** Substituting 3% of energy intake of polyunsaturated fat (PUFA) in place of vegetable protein (β = −2.94, 95% CI: −5.02, −0.86), animal protein (β = −2.68, 95% CI: −4.73, −0.63), low glycemic index (LGI) carbohydrates (β = −2.63, 95% CI: −4.40, −0.86), and high glycemic index (HGI) carbohydrates (β = −2.52, 95% CI: −4.31, −0.74) was associated with a significant reduction in SBP. Substituting 3% of the energy intake of PUFA in place of different types of carbohydrates was associated with lower odds of hypertension. PUFA was not associated with changes in DBP. **Conclusions:** Our findings suggest that exchanging PUFA for carbohydrates or proteins is associated with reduced SBP and a lower risk of hypertension, highlighting the importance of macronutrient composition independent of total energy intake and other fat types, which may have a substantial impact at the population level.

## 1. Introduction

Hypertension, or high blood pressure (HBP), is a significant global public health concern, particularly in Mexico, where its prevalence remains high (30.1%, 31.6%, and 31.5% in 2000, 2006, and 2012, respectively) [1,2,3]. According to the most recent national data, 29.9% of Mexican adults have hypertension (27.6% of women and 32.5% of men). Alarmingly, 43.0% of hypertensive adults were unaware of their condition, and only 36.3% of those diagnosed and receiving pharmacological treatment had their blood pressure under control [2,4]. HBP is a major contributor to cardiovascular diseases, and systolic blood pressure (SBP) is strongly associated with vascular mortality—each 20 mm Hg lower SBP is linked to a 48% reduction in stroke mortality and a 32% reduction in ischemic heart disease mortality. Notably, SBP was more informative for predicting vascular mortality than diastolic blood pressure (DBP) [5].

While genetic predisposition plays an important role [6], lifestyle factors, especially dietary habits, are crucial in developing and managing this condition [7,8]. Hypertension is commonly managed through pharmacological treatment and general lifestyle recommendations such as increased physical activity and sodium reduction. However, long-term adherence to medications can be challenging, and lifestyle changes are often difficult to maintain [9]. Data from the CONSTANCES cohort indicate that many individuals receiving antihypertensive treatment still fail to achieve adequate blood pressure control, largely due to modifiable lifestyle factors. These include insufficient adherence to the DASH diet, physical inactivity, excess body weight, and high alcohol consumption. These findings emphasize the need to strengthen behavioral interventions as a key component of effective hypertension management [10]. Among lifestyle factors, dietary fat intake has drawn increasing attention due to its potential role in blood pressure regulation and cardiovascular health. However, the relationship between dietary fat intake and cardiovascular health, including blood pressure (BP), remains widely debated in public health and nutrition. Historically, nutritional guidelines have emphasized reducing total fat intake to reduce the risk of cardiovascular disease (CVD) [11]. However, evidence now suggests that increasing polyunsaturated fat (PUFA) intake—especially in place of saturated fat (SFA)—appears beneficial [12]. The American Heart Association (AHA) recently reaffirmed its recommendation to replace SFA with PUFA [13]. The effects of monounsaturated fats (MUFAs) and SFA remain inconsistent, with studies showing mixed results [14,15,16,17]. Meta-analysis of observational studies showed no link between SFA intake and heart disease, while randomized trials have had mixed outcomes, suggesting that the evidence for limiting SFA may need reevaluation [18].

The specific impacts of substituting one type of fat with other macronutrients are still not well understood. In this context, it is essential to analyze macronutrient substitution, specifically examining how replacing fats with specific types of carbohydrates or proteins affects health outcomes. This analysis is crucial for clarifying the true impact of fats on cardiovascular health and other related aspects. Despite these trends, the health effects of substituting fats with other macronutrients—such as carbohydrates or proteins—remain unclear. This makes it crucial to examine not just the type of fat consumed, but also what it is replaced with. In particular, replacing fats with carbohydrates of differing glycemic index (e.g., high vs. low GI) can lead to variable effects on blood pressure and cardiovascular outcomes [19]. These differences underscore the need to assess dietary fat in the context of macronutrient substitution to understand its impact on HBP better.

The shifts in dietary patterns worldwide are particularly evident in the Mexican population, where urbanization has driven changes contributing to the rising prevalence of hypertension. Traditional Mexican diets, historically rich in complex carbohydrates such as corn, beans, vegetables, and tortillas, have transformed with urbanization and globalization. This transition has resulted in higher consumption of processed foods and unhealthy fats, particularly saturated and trans fats, commonly found in fast food and packaged snacks [20]. The nutritional transition in Mexico is characterized by the reduced consumption of fresh products and an increased intake of energy-dense foods high in sodium, sugar, and unhealthy fats [21,22]. These changes are likely contributing to the rising occurrence of HBP.

Despite these insights, limited research has specifically examined the impact of fat substitution on BP levels among Mexican adults. Given the unique dietary patterns and health challenges faced by this population, understanding how changes in specific fat intake can help manage hypertension is essential [22]. Adjusting the proportions of specific fats in the diet can influence BP over time, suggesting that strategic changes in fat intake could either increase or decrease BP levels. This study addresses these gaps by investigating how substituting different types of fats—specifically PUFA, MUFAs, and SFA—affects BP changes over time. In this longitudinal cohort study of Mexican adults, we aimed to investigate the associations between the substitution of dietary fats (PUFA, MUFAs, and SFA) with other macronutrients, particularly carbohydrates, and changes in DBP and SBP over time. We hypothesized that increasing the intake of specific types of fats, especially PUFA, over time at the expense of carbohydrates would be associated with favorable BP outcomes. Our findings provide new insights into the role of fat quality and macronutrient substitution in hypertension management within the Mexican population.

## 2. Materials and Methods

### 2.1. Study Population

This prospective study used data from the Health Workers Cohort Study (HWCS), a dynamic cohort designed to investigate the relationship between lifestyle factors and various health outcomes (Registration No. 12CEI 09 006 14) [23]. Participants were enrolled between 2004 and 2006 in Cuernavaca, Morelos, Mexico, with additional follow-up assessments conducted between 2010 and 2012 and 2017 and 2019. At baseline and during each follow-up, participants completed self-administered questionnaires providing information on sociodemographic characteristics, lifestyle habits, and medical history. A medical examination was also performed, and details regarding health status and treatments received were documented. The study protocol was approved by the Institutional Review Board at IMSS, and all participants provided written informed consent at enrollment; details have been described elsewhere [23].

**Figure 1:**
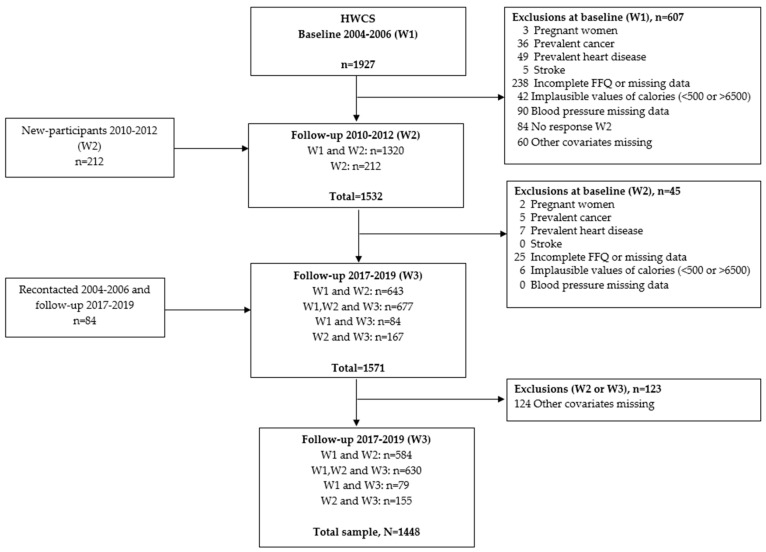
Flow chart of the participants from the Health Workers Cohort Study included in the analytic sample.

For this analysis, we included only participants who had at least two waves’ assessments with complete data. Individuals with specific medical conditions at baseline were excluded, including cancer (endometrial, ovarian, prostate, gastric, colon or rectal, lymphoma, leukemia, lung, or breast), cardiovascular disease (myocardial infarction, angina pectoris, coronary artery surgery, and stroke), and pregnant women. Participants with incomplete dietary information were also excluded—this included those who had completed less than 75% of the food frequency questionnaire (FFQ), had missing sections, or reported implausible energy intakes (i.e., less than 500 kcal/day or more than 6500 kcal/day). Additionally, participants were excluded if they had missing blood pressure or covariates data in at least two measurements. As a result, the final analysis included 1448 participants (Figure 1).

### 2.2. Dietary Assessment

Dietary information was assessed using a 116-item self-administered semi-quantitative FFQ validated for the Mexican population [24]. Participants reported the frequency and portion size of each food or beverage consumed over the past 12 months. Ten categories were used to indicate frequency: never, less than once a month, 1–3 times per month, 1, 2–4, 5–6 times per week, and 1, 2–3, 4–5, 6 or more times per day.

Macronutrients were classified into specific categories: fats were divided into three types, MUFAs, PUFA, and SFA; proteins were categorized as either vegetable or animal proteins. Additionally, total carbohydrates were categorized into two groups based on their glycemic index (GI): low glycemic index (LGI) and high glycemic index (HGI). Foods with a GI value below 50 were categorized as LGI, while those with a GI value of 50 or higher were classified as HGI.

Macronutrient and micronutrient intakes were estimated by multiplying the frequency of consumption by the portion size specified for each food item, using food composition tables provided by the National Institute of Public Health [25]. Alcohol and sodium intakes were also estimated using this method.

Total energy intake (EI) per day was calculated based on the energy content of specific macronutrients: fats (1 g = 9 kcal), carbohydrates (1 g = 4 kcal), and proteins (1 g = 4 kcal). The relative percentage of energy intake from each macronutrient was then computed using the formula:

$$\%\;of\;\mathrm{energy}\;\mathrm{from}\;\mathrm{macronutrient}=\left(\frac{EI\;\mathrm{from}\;\mathrm{macronutrient}}{\mathrm{Total}\;EI}\right)\times 100.$$

### 2.3. Blood Pressure Assessment

Trained personnel measured systolic blood pressure (SBP) and diastolic blood pressure (DBP) twice using an automated monitor (OMRON HEM-907), following established procedures and techniques [23]. The first measurement was taken after five minutes of rest, and the second measurement was obtained under the same conditions following an additional five-minute interval. The average of both measurements was used in the analysis.

Participants were classified as having hypertension if they met any of the following criteria: (1) self-reported a previous diagnosis of high blood pressure by a physician, (2) reported current use of antihypertensive medication, or (3) had a measured SBP ≥ 140 mm Hg and/or DBP ≥ 90 mm Hg, in accordance with World Health Organization (WHO) guidelines [26].

### 2.4. Covariates

Data collected included sex, age, family history of hypertension, and alcohol and sodium intake. Depressive symptoms were assessed using the Center for Epidemiologic Studies Depression Scale (CES-D) [27]. Educational level was recorded and categorized as: elementary school or less, secondary school or high school, and college or higher. Participants reported their average sleep duration during the week and on weekends, which was used to estimate their average daily hours of sleep. Physical activity (PA) was assessed using a validated questionnaire adapted for the Mexican urban population, incorporating culturally relevant leisure-time (LT) activities, such as walking, running, cycling, etc. [28]. The LT activity section included 16 items on the amount of weekly time spent performing these types of exercises. To calculate the average LT activity expended daily, time and frequency spent on each activity were added up and the total was divided by seven. LT activity was categorized as <30 min/day or ≥30 min/day based on the minimum time recommended by the World Health Organization (WHO) for maintaining health in adults [29]. Smoking status was categorized as never, former, or current smokers. Height and weight were measured following established protocols, body mass index (BMI) was calculated by dividing weight in kilograms by the square of height in meters (kg/m^2^), in accordance with WHO criteria. Type 2 diabetes (T2D) was defined as a self-reported physician-diagnosed T2D, use of hypoglycemic medication, or fasting glucose levels ≥ 126.0 mg/dL [30].

### 2.5. Statistical Analyses

We estimated the medians and percentiles for continuous variables at baseline; we also calculated the change between baseline and follow-up. To evaluate the relationship between types of fat consumption and BP changes over time, we used fixed-effects linear regression models. This approach accounts for both observed and unobserved time-invariant characteristics related to macronutrient intake and BP levels [31]. We conducted isocaloric substitution models using a 3% change unit for all macronutrients. These models estimate the effects of replacing the intake of one macronutrient with another while maintaining a constant total energy intake. Specifically, substitutions were modeled by replacing one type of fat (PUFA, MUFAs, or SFA) with either protein (separately categorized as animal or vegetable protein) or carbohydrates (categorized as low glycemic index (LGI) or high glycemic index (HGI) carbohydrates). When substituting one type of fat (e.g., PUFA) with either plant or animal protein, the model includes as covariates the percentage of carbohydrates, the other types of fats (i.e., those not being substituted), and total energy intake. The protein type used as the substitute (plant or animal) is not included as a covariate to avoid collinearity. This approach ensures that total energy intake remains constant and that the estimated effects specifically reflect the replacement of the fat type with the selected macronutrient.

Models were adjusted for potential confounders, including energy intake (kcal/day), age (years), physical activity (min/day, dichotomous), smoking status (never, former, current), alcohol intake (g/day), sleep duration (hours/day), hypertension treatment (yes/no), depressive symptoms, and sodium intake (mg/day). Sex, family history of hypertension, and education level were treated as fixed variables. Additionally, BMI was considered a mediator variable and, for this reason, was not included in the models. Statistical significance was defined as a *p*-value < 0.05. All statistical analyses were performed using Stata version 14.

Additionally, several sensitivity analyses were conducted: (1) Analysis excluding participants with diabetes or obesity at baseline, as these conditions could lead to dietary modifications during follow-up, potentially causing variable misclassification. (2) Analysis stratified by sex due to notable differences in the epidemiology and clinical characteristics of hypertension between men and women [32]. (3) An analysis stratified by hypertension status was conducted because we did not exclude prevalent cases of hypertension, where individuals might be undergoing treatment that could affect their blood pressure levels. (4) Finally an additional analysis was conducted excluding participants with diabetes, obesity, hypertension, and dyslipidemia (defined as self-reported diagnosis or use of lipid-lowering medication), since these individuals are likely to have received medical advice or treatment that could influence lifestyle-related exposures such as diet and physical activity.

We also implemented a generalized estimating equations (GEE) model using the same covariates included in the fixed-effects logistic regression, with the addition of sex, family history of hypertension, and educational level as predictors [33]. The model assumed a binomial distribution for the outcome, applied a logit link function for estimating the risk of hypertension, and used an unstructured correlation matrix to account for the relationships between repeated measurements. Unlike fixed-effects models—which focus solely on within-individual changes over time and exclude variables that do not vary within subjects (like sex)—GEE models incorporate both within- and between-subject variation. This allows for the inclusion of time-invariant covariates. While fixed-effects estimates represent purely longitudinal effects and often come with larger standard errors, GEE estimates tend to be more precise, though they reflect both intra- and inter-individual variation in the data.

## 3. Results

This analysis included 1448 participants, 73.3% women and 26.7% men at baseline. A total of 821 participants had two measurements (average follow-up time 6.7 years SD 0.97), while 627 had three measurements (average follow-up time 12.5 years SD 0.81). The median age of the participants was 45 years (P25–P75: 36–53 years), and the median BMI was 25.9 km/m^2^ (Table 1). Systolic and diastolic blood pressure increased over time, with a median change of 3 mm Hg (IQR: −7, 13) and 3 mm Hg (IQR: −4, 11), respectively. Most lifestyle variables, such as physical activity and sleep duration, showed minor changes. Regarding dietary variables, total energy intake showed a median decrease of −282 kcal/day (IQR: −799, 165). Some macronutrients showed negative median changes—such as animal protein (−2.5% of energy), animal fat (−2.5%), SFA (−0.9%), and MUFAs (−0.3%)—while others increased slightly, including vegetable protein (1.3%), vegetable fat (1.2%), and PUFA (0.2%). Carbohydrate intake also shifted modestly, with a median increase in HGI carbohydrates (0.4%) and LGI carbohydrates (1.6%) (Table 1). At baseline, 357 participants (24.7%) had hypertension; at the second measurement, 538 (37.2%); and among those who completed the third measurement, 314 (50%) had hypertension.

Using a macronutrient substitution approach, we found that replacing 3% of energy from PUFA with carbohydrates or proteins resulted in significant changes in SBP (Table 2). Specifically, PUFA intake instead of vegetable protein (SBP: β = −2.94, 95% CI: −5.02 to −9.86), animal protein (SBP: β = −2.68, 95% CI: −4.73 to −0.63), LGI carbohydrates (SBP: β = −2.63, 95% CI: −4.40 to −0.86), and HGI carbohydrates (SBP: β = −2.52, 95% CI: −4.31 to −0.74) was associated with significant reductions in SBP. Notably, no significant associations were observed for DBP. Additionally, these findings remained consistent even when participants with diabetes or obesity were excluded from the analysis (Table S1). However, substituting vegetable or animal protein, and LGI or HGI carbohydrates with SFA or MUFAs did not lead to significant changes in BP (Table 2 and Table S1).

When this additional analysis was conducted, excluding participants with diabetes, obesity, hypertension, and dyslipidemia, the associations between macronutrient substitutions and blood pressure outcomes were no longer statistically significant (Table S2).

Additionally, when we stratified the analysis by sex, significant reductions in SBP associated with substituting PUFA for various macronutrients were observed among men, but not women (Table 3).

When stratified by hypertension status at baseline, significant associations were observed between macronutrient substitutions and SBP, particularly among individuals without hypertension. Among participants without hypertension, substituting PUFA for vegetable protein (β = −2.42, 95% CI: −4.46 to −0.37), animal protein (β = −2.26, 95% CI: −4.27 to −0.24), or LGI carbohydrates (β = −1.90, 95% CI: −3.64 to −0.16) were associated with a significant reduction in SBP. Among participants with hypertension, the substitution of PUFA for LGI carbohydrates (β = −6.32, 95% CI: −11.84 to −0.81) or HGI carbohydrates (β = −6.78, 95% CI: −12.40 to −1.15) was associated with significant reductions in SBP. No significant effects were observed for DBP in any of the substitution models (Table 4).

Using generalized estimating equations (GEE), we examined the effect of substitution of different types of fats by LGI and HGI carbohydrates on hypertension risk. In the total sample, replacing LGI carbohydrates with SFA was associated with marginally reduced odds of hypertension (OR: 0.88; 95% CI: 0.76–1.00), and this association was statistically significant among females (OR: 0.83; 95% CI: 0.71–0.97), but not among males. In contrast, substituting either LGI or HGI carbohydrates with monounsaturated fats (MUFAs) was associated with increased odds of hypertension in the total sample (OR: 1.29; 95% CI: 1.09–1.53 and OR: 1.29; 95% CI: 1.09–1.52, respectively). These associations were also significant in females (OR: 1.38; 95% CI: 1.13–1.68 and OR: 1.38; 95% CI: 1.13–1.67, respectively), but not in males.

Conversely, the substitution of either LGI or HGI carbohydrates with polyunsaturated fats (PUFA) was associated with significantly lower odds of hypertension in the total sample (OR: 0.63; 95% CI: 0.50–0.80, and OR: 0.63; 95% CI: 0.49–0.80, respectively), as well as in females (OR: 0.57; 95% CI: 0.43–0.76, and OR: 0.57; 95% CI: 0.43–0.75). These associations were not statistically significant among males (Table S3).

## 4. Discussion

In this prospective study, substituting PUFA for proteins (animal or vegetable) or carbohydrates resulted in similar reductions in SBP or risk of hypertension. These effects were consistent among participants without diabetes or obesity at baseline. When stratified by sex, the fixed-effects model showed that increasing PUFA intake by replacing carbohydrates was associated with a significant decrease in systolic blood pressure (SBP) in men, but not in women. Conversely, in the generalized estimating equations (GEE) model, replacing carbohydrates with PUFA was linked to a reduced risk of hypertension in women, but not in men.

To our knowledge, this is the first study to evaluate the relationship between fat substitution and BP changes over time in Mexican adults. When reducing the intake of a particular macronutrient under isoenergetic conditions, it is necessary to compensate for this by increasing one or more of the other macronutrients. Since each macronutrient follows distinct metabolic pathways, the overall composition of the diet can significantly influence BP changes [34]. Furthermore, the health effects of macronutrients depend on their specific forms [35].

For decades, dietary guidelines emphasized that a lower total fat content equated to a healthier diet. However, researchers and public health authorities now agree that assessing the health effects of total fat intake alone is insufficient; it is essential to consider the different types of fats [18]. This study found that increasing PUFA intake instead of protein and carbohydrates was associated with decreased BP across all participants. Consistent with our findings, the INTERMAP study reported that participants with higher intakes of omega-3 PUFA acids, primarily found in fish oil, exhibited lower BP levels [36]. Additionally, the study identified an inverse relationship between linoleic acid, the predominant dietary PUFA, and BP [37]. Meta-analyses of randomized controlled trials have further demonstrated that fish oil supplementation significantly reduced BP [38].

Notably, the strongest associations reported in both previous studies and ours appear to be with SBP [38,39]. This observation may be related to the greater variability and clinical relevance of SBP in cardiovascular risk prediction, especially in middle-aged and older adults [40]. In our study, the lack of significant findings with DBP could be attributable to insufficient statistical power to detect more subtle effects on this parameter, as the magnitude of changes in DBP tends to be smaller and more difficult to capture in observational settings [38,39]. Moreover, SBP being more sensitive to arterial stiffness, a key factor in vascular aging and hypertension development [41]. Therefore, focusing on SBP may provide a more robust and clinically meaningful assessment of the impact of PUFA intake on BP regulation. This is consistent with evidence indicating that SBP is a more informative predictor of vascular mortality than DBP [5], highlighting the clinical relevance of our findings.

PUFAs are essential fats predominantly found in plant-based oils, seeds, nuts, and fatty fish. They play a crucial role in maintaining cardiovascular health and preventing hypertension through multiple mechanisms [17]. Regarding cardiovascular protection, PUFAs improve lipid profiles by lowering low-density lipoprotein (LDL) cholesterol and triglyceride levels, modulate inflammatory pathways by reducing pro-inflammatory cytokines and increasing anti-inflammatory eicosanoids, and enhance cell membrane fluidity, which supports normal cardiac and vascular function. In terms of blood pressure regulation, PUFAs contribute by improving endothelial function through increased nitric oxide production, which promotes vasodilation, and by reducing arterial stiffness and peripheral vascular resistance—key determinants of elevated systolic blood pressure [42,43,44]. Additionally, PUFAs inhibit the proliferation and migration of vascular smooth muscle cells, helping to prevent vascular remodeling and stiffness [45]. These combined effects contribute to lower blood pressure and reduce cardiovascular risk. Clinical studies support those diets rich in PUFAs are associated with improved blood pressure profiles and better cardiovascular outcomes. Clinical studies provide evidence that diets rich in PUFAs are linked to lower BP levels and better cardiovascular outcomes [46].

As previously mentioned, findings related to MUFAs and SFA remain contradictory [14,15,16,17]. The literature presents mixed evidence, with some studies reporting benefits and others suggesting potential risks. For example, Jakobsen et al. found that replacing saturated fatty acids (SFAs) with polyunsaturated fatty acids (PUFAs) or cis-monounsaturated fatty acids (MUFAs) was associated with a reduced risk of coronary heart disease [47]. Conversely, the PURE study by Dehghan et al. (2017) reported that higher intakes of SFAs were linked to lower mortality and stroke risk, challenging conventional dietary guidelines [16]. These contradictory findings highlight the importance of considering substitution nutrients, population characteristics, and dietary patterns when evaluating fat intake and cardiovascular risk.

In the fixed effects model, no association was observed between MUFA intake and SBP or DBP, suggesting no within-individual effect over time. In contrast, the generalized estimating equations (GEE) model—designed to estimate population-averaged effects—showed an increased risk of hypertension when carbohydrates were replaced with MUFAs, particularly among women. It is important to note that GEE models are more sensitive to between-individual variation and may be influenced by residual confounding, which could explain discrepancies between the two models. This finding suggests that increasing MUFA intake—specifically when replacing LGI or HGI carbohydrates—may be associated with a higher risk of hypertension. Supporting this, animal studies have shown that LDL receptor knockout mice fed diets rich in MUFAs developed more atherosclerosis compared to those fed diets high in PUFA or SFA [48]. A meta-analysis of randomized controlled trials has shown that replacing carbohydrates with MUFAs did not significantly affect BP in the short-term [14].

Additionally, a preliminary study reported that a single MUFA-rich meal—specifically from oleic acid—impaired postprandial endothelial function compared to an isoenergetic carbohydrates-rich meal [49]. It has also been suggested that a MUFA intake exceeding 20% of total energy may be required to observe beneficial effects on BP [50]. Interestingly, when modeling the substitution of SFA with MUFAs, the association with hypertension was no longer statistically significant (OR: 1.25, 95% CI: 0.90–1.73), suggesting that the adverse effect may depend on the specific macronutrient being replaced. These findings highlight the complexity of macronutrient interactions and underscore the need for further studies to better understand the long-term effects of MUFA substitution on BP, particularly within different dietary patterns and population subgroups. On the other hand, we found an increase in SFA intake, which, when substituted for LGI or HGI carbohydrates, showed a protective effect against hypertension in the GEE model, particularly among women. However, this association was not observed in the fixed effects model, where no relationship with SBP or DBP was found. A cross-sectional study supported this finding, but did not include specific substitution analyses [51]. While current evidence suggests that replacing SFA with PUFAs may reduce the risk of cardiovascular events, substitution with carbohydrates has no significant effect on coronary heart disease (CHD) or mortality [15]. A longitudinal study even found a protective effect of SFA intake against ischemic heart disease. Furthermore, another longitudinal study using an isocaloric substitution model reported that increasing animal protein, cis-MUFAs, PUFA, or carbohydrate intake—at the expense of reducing SFA—was associated with a higher risk of ischemic heart disease [52]. These findings suggest that recommending SFA reduction in isolation may not yield cardiovascular benefits unless the replacement nutrients are carefully considered. Therefore, more longitudinal and interventional studies are needed to clarify the effects of MUFAs and SFA substitution across different dietary contexts and populations.

Furthermore, when we evaluated the relationship of interest according to sex, we found that the associations were observed only in men using the fixed effects model. In contrast, in women, the associations emerged under the GEE model. Studies have shown that men and women may respond differently to dietary interventions due to differences in body composition, hormonal fluctuations, and metabolic rates [32]. For instance, men generally have higher visceral fat, which is more metabolically active and could influence BP responses differently than women [53]. Moreover, in a similar study evaluating the relationship between macronutrient substitution and metabolic syndrome, a significant decrease in BP was noted only for men, when their total fat intake was increased over carbohydrates or alcohol intake [34]. Hormonal differences may have partially mediated these sex-specific responses; for example, estrogen has vasodilatory and anti-inflammatory effects that may modulate the impact of dietary fats on BP regulation in women [54]. Menopausal status may further influence these associations, as the decline in estrogen levels post-menopause has been linked to increased vascular stiffness and altered lipid metabolism [55]. These findings highlight the need for sex-specific approaches in dietary guidelines, particularly concerning fat intake and cardiovascular risk management.

In this study, we observed differences in the effect of PUFA substitution on SBP between hypertensive participants and those who were normotensive at baseline, with more pronounced effects among those with hypertension. These differences may be explained by distinct physiological responses to macronutrient changes, particularly about baseline health status and inflammatory profiles. Hypertensive individuals, who often present with elevated inflammation, oxidative stress, and endothelial dysfunction [56,57], may derive greater benefit from the anti-inflammatory and vascular-protective properties of PUFA. In contrast, normotensive individuals, who typically have lower baseline vascular stress, may experience more modest BP changes in response to dietary fat modification [17].

This study had several strengths, including a prospective design with a broad age range and including both sexes. Using at least two FFQ assessments enhanced the accuracy and precision of dietary measurements, allowing us to estimate changes in macronutrient intake over time. We evaluated several types of fats, as their structural differences exhibited different effects on BP. Additionally, the use of fixed effects models helped minimize the impact of confounding factors due to time-invariant factors or unmeasured characteristics but have time-invariant effects. However, there are some limitations to consider. As an observational study, we cannot eliminate residual confounding, even after adjusting for major risk factors for hypertension. Furthermore, since diet intake was self-reported, measurement errors could occur, which may lead to some participants being misclassified regarding their dietary exposure. Lastly, the study was conducted in an urban population from central Mexico, so its findings may not be generalizable to other populations.

## 5. Conclusions

The results from this study offer new insights, particularly for Latin American populations, where research on the relationship between dietary intake and blood pressure is limited. Our findings consistently indicate that prioritizing fats, especially those from vegetable, animal, and PUFA sources, over carbohydrates or proteins is associated with lower SBP. While previous studies have shown mixed results, our data support the beneficial role of specific macronutrient substitutions in managing hypertension. Importantly, we highlight the need to tailor dietary recommendations according to individual factors such as sex and hypertension status to optimize intervention outcomes. Overall, these results reinforce the importance of balanced dietary strategies emphasizing quality fat intake for preventing and managing hypertension in diverse populations. Practical dietary recommendations include increasing the consumption of foods rich in PUFAs such as avocados, nuts, seeds, and fatty fish such as salmon, while reducing the intake of carbohydrates like white bread, sugary snacks, and sweetened beverages. Encouraging both men and women to include these foods regularly can support cardiovascular health and blood pressure control.

## Figures and Tables

**Table 1:** Baseline characteristics and changes in the Health Workers Cohort Study (HWCS) (n = 1448).

Characteristics	Baseline	Changes
	Median (p25, p75)	Median (p25, p75)
Age (years)	45 (36, 53)	7 (6, 10)
Systolic Blood pressure (mm Hg)	115 (108, 123)	3 (−7, 13)
Diastolic Blood pressure (mm Hg)	71 (65, 78)	3 (−4, 11)
Sleep time (hours/day)	7.3 (6.6, 8.0)	0 (−0.71, 0.71)
Physical activity (min/day)	12.9 (3.2, 34.9)	0 (−15.6, 11.4)
BMI (kg/m^2^)	25.9 (23.5, 28.8)	0.6 (−0.7, 2.0)
Depressive symptoms	14 (12, 19)	0 (−4, 4)
Dietary variables		
Total energy (kcal/day)	1921 (1473, 2478)	−282 (−799, 165)
Animal protein (% of energy)	14.9 (11.6, 18.5)	−2.5 (−6.7, 1.6)
Vegetal protein (% of energy)	8.6 (7.0, 10.5)	1.3 (−1.1, 4.0)
Animal fat (% of energy)	14.9 (11.6, 18.5)	−2.5 (−6.7, 1.6)
Vegetal fat (% of energy)	8.6 (7.0, 10.5)	1.2 (−1.0, 3.5)
Saturated fat (% of energy)	8.6 (7.1, 10.4)	−0.9 (−2.8, 1.0)
Monounsaturated fat (% of energy)	9.8 (8.3, 11.6)	−0.3 (−2.4, 1.7)
Polyunsaturated fat (% of energy)	4.2 (3.6, 5.0)	0.2 (−0.6, 1.2)
High glycemic index carbohydrate (% of energy)	43.2 (37.1, 49.1)	0.4 (−7.0, 7.6)
Low glycemic index carbohydrate (% of energy)	17.7 (13.6, 22.9)	1.6 (−3.5, 6.5)
Alcohol (g/day)	1.0 (0.2, 4.0)	0 (−0.9, 0.6)
Sodium (g/day)	1.7 (1.3, 2.3)	−0.3 (−0.8, 0.2)

**Table 2:** Association between dietary fat substitution intake and changes in blood pressure. Health Workers Cohort Study 2004–2018.

	Macronutrients Intake		SBP	DBP
	Increased	Decreased	β (IC95%)	β (IC95%)
Unadjusted	Saturated fat	Vegetal Protein	1.17 (−0.76, 3.09)	0.46 (−1.93, 2.86)
Adjusted	Saturated fat	Vegetal Protein	0.16 (−1.80, 2.11)	−0.43 (−3.00, 2.13)
Unadjusted	Monounsaturated fat	Vegetal Protein	0.48 (−1.13, 2.09)	0.51 (−1.49, 2.52)
Adjusted	Monounsaturated fat	Vegetal Protein	0.02 (−1.64, 1.69)	0.02 (−2.16, 2.20)
Unadjusted	Polyunsaturated fat	Vegetal Protein	−0.57 (−2.58, 1.43)	2.91 (0.42, 5.41) *
Adjusted	Polyunsaturated fat	Vegetal Protein	−2.94 (−5.02, −0.86) **	0.79 (−1.93, 3.50)
Unadjusted	Saturated fat	Animal Protein	−0.31 (−1.95, 1.34)	−0.13 (−2.17, 1.90)
Adjusted	Saturated fat	Animal Protein	−0.86 (−2.56, 0.84)	−0.68 (−2.91, 1.54)
Unadjusted	Monounsaturated fat	Animal Protein	−0.27 (−2.56, 2.02)	0.16 (−2.69, 3.00)
Adjusted	Monounsaturated fat	Animal Protein	0.98 (−1.36, 3.31)	1.24 (−1.82, 4.29)
Unadjusted	Polyunsaturated fat	Animal Protein	−0.17 (−2.17, 1.82)	3.08 (0.60, 5.55)*
Adjusted	Polyunsaturated fat	Animal Protein	−2.68 (−4.73, −0.63) *	0.79 (−1.90, 3.48)
Unadjusted	Saturated fat	LGI Carbohydrate	−2.08 (−3.13, −1.04) **	−1.78 (−3.08, −0.49) **
Adjusted	Saturated fat	LGI Carbohydrate	−0.57 (−1.65, 0.51)	−0.41 (−1.82, 1.00)
Unadjusted	Monounsaturated fat	LGI Carbohydrate	0.33 (−0.96, 1.62)	−0.19 (−1.79, 1.41)
Adjusted	Monounsaturated fat	LGI Carbohydrate	0.50 (−0.77, 1.78)	−0.12 (−1.79, 1.54)
Unadjusted	Polyunsaturated fat	LGI Carbohydrate	−1.05 (−2.84, 0.74)	1.97 (−0.26, 4.19)
Adjusted	Polyunsaturated fat	LGI Carbohydrate	−2.63 (−4.40, −0.86) **	0.59 (−1.72, 2.91)
Unadjusted	Saturated fat	HGI Carbohydrate	−1.55 (−2.54, −0.56) **	−1.79 (−3.02, −0.56) **
Adjusted	Saturated fat	HGI Carbohydrate	−0.42 (−1.44, 0.59)	−0.71 (−2.04, 0.62)
Unadjusted	Monounsaturated fat	HGI Carbohydrate	0.72 (−0.55, 1.98)	−0.20 (−1.77, 1.36)
Adjusted	Monounsaturated fat	HGI Carbohydrate	0.61 (−0.63, 1.86)	−0.36 (−1.99, 1.27)
Unadjusted	Polyunsaturated fat	HGI Carbohydrate	−0.61 (−2.40, 1.19)	2.00 (−0.23, 4.23)
Adjusted	Polyunsaturated fat	HGI Carbohydrate	−2.52 (−4.31, −0.74) **	0.33 (−2.01, 2.66)

SBP: Systolic blood pressure. DBP: Diastolic blood pressure. LGI: Low glycemic index carbohydrate. HGI: High glycemic index carbohydrate. Models referred to as “unadjusted” were adjusted for total energy intake (kcal/day continuous) and included all macronutrients except the one being replaced, as per the isocaloric substitution approach. The “adjusted” models included all the variables from the “unadjusted” models (total energy intake and macronutrients except the one being replaced) as well as additional covariates: age (years, continuous), physical activity (minutes/day, dichotomous), smoking (never, former, and current), alcohol intake (g/day, continuous), sleep duration (hours/day, continuous), treatment for hypertension (yes/no), depressive symptoms (continuous), and sodium intake (mg/day, continuous). Isocaloric substitution models—with a unit of change of 3% for all the macronutrients. When evaluating specific types of macronutrients, the rest of the macronutrients were included in the model. * *p* < 0.05, ** *p* < 0.01.

**Table 3:** Association between dietary fat substitution intake and changes in blood pressure by sex.

			Males, n = 387		Females, n = 1060	
	Macronutrients Intake		SBP	DBP	SBP	DBP
	Increased	Decreased	β (IC95%)	β (IC95%)	β (IC95%)	β (IC95%)
Unadjusted	Saturated fat	Vegetal Protein	4.14 (−0.07, 8.36)	2.00 (−1.70, 5.71)	0.58 (−1.58, 2.75)	0.05 (−2.89, 2.98)
Adjusted	Saturated fat	Vegetal Protein	1.87 (−2.25, 5.98)	1.01 (−2.69, 4.70)	−0.17 (−2.41, 2.06)	−0.74 (−3.95, 2.47)
Unadjusted	Monounsaturated fat	Vegetal Protein	4.02 (−0.11, 8.15)	2.03 (−1.60, 5.67)	−0.22 (−1.97, 1.52)	0.27 (−2.10, 2.64)
Adjusted	Monounsaturated fat	Vegetal Protein	3.29 (−0.74, 7.31)	1.83 (−1.78, 5.44)	−0.59 (−2.43, 1.25)	−0.27 (−2.91, 2.37)
Unadjusted	Polyunsaturated fat	Vegetal Protein	−3.14 (−7.42, 1.13)	1.16 (−2.59, 4.92)	0.21 (−2.06, 2.48)	3.58 (0.50, 6.66) *
Adjusted	Polyunsaturated fat	Vegetal Protein	−6.57 (−10.96, 2.17) **	−1.72 (−5.66, 2.22)	−1.80 (−4.16, 0.57)	1.82 (−1.57, 5.21)
Unadjusted	Saturated fat	Animal Protein	2.24 (−1.46, 5.94)	1.22 (−2.03, 4.47)	−0.72 (−2.55, 1.11)	−0.51 (−2.99, 1.97)
Adjusted	Saturated fat	Animal Protein	0.65 (−3.06, 4.36)	0.70 (−2.63, 4.03)	−1.01 (−2.93, 0.91)	−1.07 (−3.82, 1.68)
Unadjusted	Monounsaturated fat	Animal Protein	5.07 (−0.08, 10.21)	0.64 (−3.88, 5.16)	−1.70 (−4.28, 0.87)	0.34 (−3.15, 3.83)
Adjusted	Monounsaturated fat	Animal Protein	6.19 (1.26, 11.12) *	2.37 (−2.06, 6.81)	−0.50 (−3.19, 2.19)	1.22 (−2.63, 5.07)
Unadjusted	Polyunsaturated fat	Animal Protein	−2.42 (−6.64, 1.81)	1.39 (−2.32, 5.10)	0.55 (−1.71, 2.81)	3.72 (0.66, 6.78) *
Adjusted	Polyunsaturated fat	Animal Protein	−6.09 (−10.38, −1.80) **	−1.59 (−5.45, 2.26)	−1.59 (−3.93, 0.76)	1.81 (−1.55, 5.17)
Unadjusted	Saturated fat	LGI Carbohydrate	−0.75 (−3.27, 1.77)	−2.87 (−5.09, 0.64) *	−2.42 (−3.57,1.27) **	−1.45 (−3.00, 0.10)
Adjusted	Saturated fat	LGI Carbohydrate	0.81 (−1.67, 3.29)	−0.91 (−3.14, 1.32)	−1.00 (−2.21, 0.20)	−0.35 (−2.08, 1.37)
Unadjusted	Monounsaturated fat	LGI Carbohydrate	1.66 (−1.56, 4.89)	−0.98 (−3.82, 1.87)	−0.04 (−1.44, 1.37)	−0.09 (−1.99, 1.81)
Adjusted	Monounsaturated fat	LGI Carbohydrate	3.00 (−0.06, 6.05)	0.32 (−2.42, 3.07)	−0.06 (−1.46, 1.34)	−0.18 (−2.19, 1.83)
Unadjusted	Polyunsaturated fat	LGI Carbohydrate	−4.48 (−8.40, −0.57) *	−0.60 (−4.05, 2.86)	−0.06 (−2.08, 1.96)	2.96 (0.23, 5.69) *
Adjusted	Polyunsaturated fat	LGI Carbohydrate	−6.97 (−10.74, −3.19) **	−3.10 (−6.49, 0.29)	−1.49 (−3.51, 0.53)	1.93 (−0.97, 4.83)
Unadjusted	Saturated fat	HGI Carbohydrate	−0.60 (−2.95, 1.76)	−1.84 (−3.91, 0.24)	−1.82 (−2.91, −0.72)	−1.75 (−3.24, −0.27) *
Adjusted	Saturated fat	HGI Carbohydrate	0.35 (−1.94, 2.64)	−0.50 (−2.56, 1.56)	−0.69 (−1.83, 0.44)	−0.85 (−2.47, 0.78)
Unadjusted	Monounsaturated fat	HGI Carbohydrate	1.86 (−1.30, 5.02)	0.02 (−2.75, 2.80)	0.38 (−0.99, 1.75)	−0.31 (−2.16, 1.54)
Adjusted	Monounsaturated fat	HGI Carbohydrate	2.60 (−0.40, 5.61)	0.73 (−1.96, 3.43)	0.18 (−1.20, 1.55)	−0.56 (−2.52, 1.41)
Unadjusted	Polyunsaturated fat	HGI Carbohydrate	−4.20 (−8.14, −0.25) *	0.26 (−3.20, 3.73)	0.43 (−1.59, 2.45)	2.73 (0.00, 5.46)
Adjusted	Polyunsaturated fat	HGI Carbohydrate	−7.29 (−11.11, −3.47) **	−2.67 (−6.10, 0.76)	−1.24 (−3.28, 0.79)	1.48 (−1.44, 4.39)

SBP: Systolic blood pressure. DBP: Diastolic blood pressure. LGI: Low glycemic index carbohydrate. HGI: High glycemic index carbohydrate. Models referred to as “unadjusted” were adjusted for total energy intake (kcal/day continuous), and included all macronutrients except the one being replaced, as per the isocaloric substitution approach. The “adjusted” models included all the variables from the “unadjusted” models (total energy intake and macronutrients except the one being replaced) as well as additional covariates: age (years, continuous), physical activity (minutes/day, dichotomous), smoking (never, former, and current), alcohol intake (g/day, continuous), sleep duration (hours/day, continuous), treatment for hypertension (yes/no), depressive symptoms (continuous), and sodium intake (mg/day, continuous). Isocaloric substitution models—with a unit of change of 3% for all the macronutrients. When evaluating specific types of macronutrients, the rest of the macronutrients were included in the model. * *p* < 0.05, ** *p* < 0.01.

**Table 4:** Association between dietary macronutrient substitution intake and changes in blood pressure according to hypertension status at baseline.

			Without Hypertension, n = 1090		With Hypertension, n = 357	
	Macronutrients Intake		SBP	DBP	SBP	DBP
	Increased	Decreased	β (IC95%)	β (IC95%)	β (IC95%)	β (IC95%)
Unadjusted	Saturated fat	Vegetal Protein	−0.57 (−2.54, 1.41)	−0.34 (−3.26, 2.58)	6.31 (1.34, 11.27) *	1.50 (−2.47, 5.47)
Adjusted	Saturated fat	Vegetal Protein	−0.72 (−2.74, 1.29)	−0.66 (−3.78, 2.47)	2.47 (−2.77, 7.72)	0.30 (−4.09, 4.70)
Unadjusted	Monounsaturated fat	Vegetal Protein	−0.16 (−1.86, 1.54)	−0.30 (−2.82, 2.21)	1.88 (−2.08, 5.85)	2.77 (−0.40, 5.94)
Adjusted	Monounsaturated fat	Vegetal Protein	−0.79 (−2.54, 0.96)	−1.39 (−4.11, 1.32)	2.56 (−1.67, 6.79)	3.75 (0.21, 7.30) *
Unadjusted	Polyunsaturated fat	Vegetal Protein	−0.51 (−2.49, 1.47)	2.44 (−0.50, 5.38)	−1.65 (−7.59, 4.29)	3.88 (−0.86, 8.63)
Adjusted	Polyunsaturated fat	Vegetal Protein	−2.42 (−4.46, −0.37) *	0.20 (−2.98, 3.37)	−5.13 (−11.58, 1.32)	2.84 (−2.57, 8.24)
Unadjusted	Saturated fat	Animal Protein	−1.39 (−3.08, 0.30)	−1.17 (−3.68, 1.34)	2.80 (−1.34, 6.95)	1.96 (−1.34, 5.25)
Adjusted	Saturated fat	Animal Protein	−1.25 (−3.02, 0.51)	−1.24 (−3.98, 1.50)	0.27 (−4.09, 4.63)	1.40 (−2.26, 5.06)
Unadjusted	Monounsaturated fat	Animal Protein	−1.26 (−3.60, 1.08)	−0.87 (−4.34, 2.60)	2.25 (−3.72, 8.22)	3.04 (−1.71, 7.79)
Adjusted	Monounsaturated fat	Animal Protein	−0.41 (−2.80, 1.98)	−0.02 (−3.72, 3.69)	5.55 (−0.67, 11.77)	4.73 (−0.49, 9.94)
Unadjusted	Polyunsaturated fat	Animal Protein	−0.24 (−2.20, 1.71)	2.70 (−0.20, 5.60)	−1.08 (−7.04, 4.89)	3.83 (−0.91, 8.58)
Adjusted	Polyunsaturated fat	Animal Protein	−2.26 (−4.27, −0.24)	0.30 (−2.82, 3.43)	−4.86 (−11.31, 1.59)	2.79 (−2.62, 8.20)
Unadjusted	Saturated fat	LGI Carbohydrate	−2.40 (−3.49, −1.31) *	−2.04 (−3.65, −0.43) *	−1.21 (−3.79, 1.37)	−1.07 (−3.12, 0.97)
Adjusted	Saturated fat	LGI Carbohydrate	−0.67 (−1.82, 0.48)	0.02 (−1.76, 1.80)	−0.35 (−2.96, 2.25)	−0.96 (−3.15, 1.22)
Unadjusted	Monounsaturated fat	LGI Carbohydrate	−0.07 (−1.45, 1.31)	−0.31 (−2.35, 1.74)	1.93 (−1.16, 5.03)	0.14 (−2.31, 2.59)
Adjusted	Monounsaturated fat	LGI Carbohydrate	−0.12 (−1.48, 1.24)	−0.61 (−2.72, 1.50)	2.78 (−0.38, 5.95)	0.63 (−2.02, 3.29)
Unadjusted	Polyunsaturated fat	LGI Carbohydrate	−0.52 (−2.28, 1.25)	2.30 (−0.31, 4.91)	−4.02 (−9.36, 1.32)	1.14 (−3.09, 5.38)
Adjusted	Polyunsaturated fat	LGI Carbohydrate	−1.90 (−3.64, −0.16) *	0.92 (−1.77, 3.61)	−6.32 (−11.84, −0.81) *	−0.13 (−4.77, 4.50)
Unadjusted	Saturated fat	HGI Carbohydrate	−1.58 (−2.62, −0.54)	−2.29 (−3.83, −0.75) *	−1.58 (−4.00, 0.84)	−0.50 (−2.42, 1.41)
Adjusted	Saturated fat	HGI Carbohydrate	−0.25 (−1.34, 0.83)	−0.63 (−2.30, 1.05)	−1.01 (−3.44, 1.42)	−0.47 (−2.51, 1.57)
Unadjusted	Monounsaturated fat	HGI Carbohydrate	0.56 (−0.79, 1.91)	−0.50 (−2.50, 1.49)	1.65 (−1.36, 4.66)	0.52 (−1.87, 2.90)
Adjusted	Monounsaturated fat	HGI Carbohydrate	0.21 (−1.13, 1.54)	−1.14 (−3.20, 0.93)	2.31 (−0.77, 5.39)	0.97 (−1.61, 3.55)
Unadjusted	Polyunsaturated fat	HGI Carbohydrate	0.10 (−1.67, 1.86)	2.12 (−0.49, 4.74)	−4.03 (−9.44, 1.37)	1.76 (−2.52, 6.04)
Adjusted	Polyunsaturated fat	HGI Carbohydrate	−1.60 (−3.35, 0.14)	0.34 (−2.36, 3.05)	−6.78 (−12.40, −1.15) *	0.49 (−4.22, 5.21)

SBP: Systolic blood pressure. DBP: Diastolic blood pressure. LGI: Low glycemic index carbohydrate. HGI: High glycemic index carbohydrate. Models referred to as “unadjusted” were adjusted for total energy intake (kcal/day continuous) and included all macronutrients except the one being replaced, as per the isocaloric substitution approach. The “adjusted” models included all the variables from the “unadjusted” models (total energy intake and macronutrients except the one being replaced) as well as additional covariates: age (years, continuous), physical activity (minutes/day, dichotomous), smoking (never, former, and current), alcohol intake (g/day, continuous), sleep duration (hours/day, continuous), treatment for hypertension (yes/no), depressive symptoms (continuous), and sodium intake (mg/day, continuous). * *p* < 0.05.

## Data Availability

The datasets used and analyzed during in this study are available at this link: https://drive.google.com/drive/u/1/folders/1Drlgv_YsYfC-yToAgtidT9rlN3QmBDKW (accessed on 19 June 2025).

## Supplementary Materials

The following supporting information can be downloaded at: https://www.mdpi.com/article/10.3390/nu17132096/s1, Table S1. Association between dietary fat substitution intake and changes in blood pressure in participants free of diabetes or obesity at baseline. Health Workers Cohort Study 2004–2018 (n = 1292); Table S2. Association between dietary fat substitution intake and arterial hypertension in the Health Workers Cohort Study 2004–2018; Table S3. Association between dietary fat substitution intake and arterial hypertension in the Health Workers Cohort Study 2004–2018.

## Abbreviations

The following abbreviations are used in this manuscript:

HBP	High Blood Pressure
SBP	Systolic Blood Pressure
DBP	Diastolic Blood Pressure
CVD	Cardiovascular Disease
PUFA	Polyunsaturated Fat
AHA	American Heart Association
SFA	Saturated Fat
MUFA	Monounsaturated Fat
HWCS	Health Workers Cohort Study
FFQ	Food Frequency Questionnaire
GI	Glycemic Index
LGI	Low Glycemic Index
HGI	High Glycemic Index
EI	Energy Intake
WHO	World Health Organization
PA	Physical Activity
BMI	Body Mass Index
T2D	Type 2 Diabetes
GEE	Generalized Estimating Equations
