# Supplementary material for: Dietary Fats Substitution and Blood Pressure Levels: A Longitudinal Study in Mexican Adults
Source: Nutrients. 2025 Jun 24;17(13):2096. doi: 10.3390/nu17132096 (PMC12251371; doi:10.3390/nu17132096)
Supplement: Supplementary file 1 [file nutrients-17-02096-s001.zip › nutrients-3687686-supplementary.pdf]

Supplementary Table S1. Association between dietary fat substitution intake and changes in blood pressure in participants free of diabetes or obesity at baseline. Health Workers Cohort Study 2004-2018 (n=1292).

	**Macronutrients intake**		**SBP**	**DBP**
	**Increased**	**Decreased**	**β(IC95%)**	**β(IC95%)**
Unadjusted	Saturated fat	Vegetal Protein	0.77 (-1.19, 2.73)	-0.01 (-2.63, 2.60)
Adjusted	Saturated fat	Vegetal Protein	0.20 (-1.83, 2.24)	-0.83 (-3.64, 1.97)
Unadjusted	Monounsaturated fat	Vegetal Protein	0.41 (-1.25, 2.07)	0.29 (-1.92, 2.50)
Adjusted	Monounsaturated fat	Vegetal Protein	0.12 (-1.62, 1.86)	-0.19 (-2.58, 2.21)
Unadjusted	Polyunsaturated fat	Vegetal Protein	-0.26 (-2.32, 1.80)	3.40 (0.66, 6.14)*
Adjusted	Polyunsaturated fat	Vegetal Protein	-2.20 (-4.36,-0.03)*	1.15 (-1.84, 4.14)
Unadjusted	Saturated fat	Animal Protein	-0.54 (-2.22, 1.14)	-0.44 (-2.67, 1.79)
Adjusted	Saturated fat	Animal Protein	-0.78 (-2.55, 1.00)	-0.99 (-3.44, 1.45)
Unadjusted	Monounsaturated fat	Animal Protein	-0.57 (-2.92, 1.77)	0.03 (-3.08, 3.15)
Adjusted	Monounsaturated fat	Animal Protein	0.72 (-1.71, 3.14)	1.22 (-2.11, 4.56)
Unadjusted	Polyunsaturated fat	Animal Protein	0.10 (-1.94, 2.14)	3.52 (0.81, 6.23)*
Adjusted	Polyunsaturated fat	Animal Protein	-1.91 (-4.05, 0.23)	1.12 (-1.83, 4.07)
Unadjusted	Saturated fat	LGI Carbohydrate	-2.15 (-3.23,-1.08)**	-1.89 (-3.32,-0.46)*
Adjusted	Saturated fat	LGI Carbohydrate	-0.85 (-1.98, 0.29)	-0.50 (-2.06, 1.06)
Unadjusted	Monounsaturated fat	LGI Carbohydrate	0.35 (-0.99, 1.69)	-0.43 (-2.21, 1.34)
Adjusted	Monounsaturated fat	LGI Carbohydrate	0.43 (-0.91, 1.77)	-0.34 (-2.18, 1.50)
Unadjusted	Polyunsaturated fat	LGI Carbohydrate	-0.64 (-2.48, 1.20)	2.48 (0.03, 4.92)*
Adjusted	Polyunsaturated fat	LGI Carbohydrate	-2.08 (-3.93,-0.24*	0.97 (-1.57, 3.51)

Unadjusted	Saturated fat	HGI Carbohydrate	-1.67 (-2.70,-0.65)**	-1.92 (-3.28,-0.55)**
Adjusted	Saturated fat	HGI Carbohydrate	-0.72 (-1.79, 0.35)	-0.81 (-2.28, 0.67)
Unadjusted	Monounsaturated fat	HGI Carbohydrate	0.70 (-0.61, 2.00)	-0.47 (-2.20, 1.27)
Adjusted	Monounsaturated fat	HGI Carbohydrate	0.53 (-0.78, 1.84)	-0.58 (-2.38, 1.22)
Unadjusted	Polyunsaturated fat	HGI Carbohydrate	-0.25 (-2.09, 1.60)	2.48 (0.04, 4.93)*
Adjusted	Polyunsaturated fat	HGI Carbohydrate	-1.98 (-3.84,-0.12)*	0.69 (-1.87, 3.25)

SBP: Systolic blood pressure. LGI: Low glycemic index carbohydrate- HGI: High glycemic index carbohydrate. Models referred to as "unadjusted" were adjusted for total energy intake (kcal/day continuous) and included all macronutrients except the one being replaced, as per the isocaloric substitution approach. The "adjusted" models included all the variables from the "unadjusted" models (total energy intake and macronutrients except the one being replaced) as well as additional covariates: age (years, continuous), physical activity (minutes/day, dichotomous), smoking (never, former, and current), alcohol intake (g/day, continuous), sleep duration (hours/day, continuous), treatment for hypertension (yes/no), depressive symptoms (continuous), and sodium intake (mg/day, continuous). Isocaloric substitution models- with a unit of change of 3% for all the macronutrients. When evaluating specific types of macronutrients, the rest of the macronutrients were included in the model. * $p < 0.05$- ** $p<0.01$.

Supplementary Table S2. Association between dietary fat substitution intake and changes in blood pressure in participants free of diabetes, obesity, hypertension, or dyslipidemia at baseline. Health Workers Cohort Study 2004-2018 (n=561).

	Macronutrients intake		SBP	DBP
	**Increased**	**Decreased**	**β(IC95%)**	**β(IC95%)**
**Unadjusted**	Saturated fat	Vegetal Protein	-0.59 (-3.35, 2.18)	-1.23 (-6.72, 4.25)
**Adjusted**	Saturated fat	Vegetal Protein	-0.69 (-3.49, 2.10)	-1.36 (-7.03, 4.30)
**Unadjusted**	Monounsaturated fat	Vegetal Protein	-0.34 (-2.77, 2.08)	0.35 (-4.46, 5.16)
**Adjusted**	Monounsaturated fat	Vegetal Protein	-1.28 (-3.83, 1.26)	-1.11 (-6.27, 4.05)
**Unadjusted**	Polyunsaturated fat	Vegetal Protein	1.99 (-0.77, 4.74)	4.82 (-0.65,10.30)
**Adjusted**	Polyunsaturated fat	Vegetal Protein	-0.43 (-3.45, 2.60)	1.79 (-4.34, 7.92)
**Unadjusted**	Saturated fat	Animal Protein	-1.05 (-3.40, 1.30)	-2.62 (-7.27, 2.04)
**Adjusted**	Saturated fat	Animal Protein	-1.23 (-3.68, 1.22)	-2.50 (-7.46, 2.45)
**Unadjusted**	Monounsaturated fat	Animal Protein	-1.06 (-4.34, 2.22)	1.12 (-5.38, 7.63)
**Adjusted**	Monounsaturated fat	Animal Protein	-0.57 (-3.97, 2.83)	1.97 (-4.91, 8.84)
**Unadjusted**	Polyunsaturated fat	Animal Protein	2.11 (-0.63, 4.84)	5.17 (-0.25,10.59)
**Adjusted**	Polyunsaturated fat	Animal Protein	-0.28 (-3.25, 2.70)	1.97 (-4.05, 7.99)
**Unadjusted**	Saturated fat	LGI Carbohydrate	-2.17 (-3.74,-0.60)*	-2.84 (-5.95, 0.27)
**Adjusted**	Saturated fat	LGI Carbohydrate	-0.82 (-2.49, 0.84)	-0.42 (-3.79, 2.96)
**Unadjusted**	Monounsaturated fat	LGI Carbohydrate	-0.66 (-2.65, 1.34)	0.48 (-3.47, 4.44)
**Adjusted**	Monounsaturated fat	LGI Carbohydrate	-0.85 (-2.86, 1.16)	0.01 (-4.07, 4.08)
**Unadjusted**	Polyunsaturated fat	LGI Carbohydrate	1.42 (-1.08, 3.92)	4.64 (-0.31, 9.60)
**Adjusted**	Polyunsaturated fat	LGI Carbohydrate	-0.15 (-2.71, 2.41)	2.85 (-2.34, 8.05)

**Unadjusted**	Saturated fat	HGI Carbohydrate	-1.53 (-3.05,-0.01)*	-3.85 (-6.86,-0.83)
**Adjusted**	Saturated fat	HGI Carbohydrate	-0.45 (-2.03, 1.13)	-1.79 (-5.00, 1.42)
**Unadjusted**	Monounsaturated fat	HGI Carbohydrate	-0.13 (-2.07, 1.81)	-0.36 (-4.21, 3.50)
**Adjusted**	Monounsaturated fat	HGI Carbohydrate	-0.53 (-2.50, 1.43)	-1.15 (-5.12, 2.83)
**Unadjusted**	Polyunsaturated fat	HGI Carbohydrate	1.93 (-0.58, 4.43)	3.86 (-1.11, 8.83)
**Adjusted**	Polyunsaturated fat	HGI Carbohydrate	0.15 (-2.43, 2.74)	1.52 (-3.71, 6.76)

SBP: Systolic blood pressure. LGI: Low glycemic index carbohydrate- HGI: High glycemic index carbohydrate. Models referred to as "unadjusted" were adjusted for total energy intake (kcal/day continuous) and included all macronutrients except the one being replaced, as per the isocaloric substitution approach. The "adjusted" models included all the variables from the "unadjusted" models (total energy intake and macronutrients except the one being replaced) as well as additional covariates: age (years, continuous), physical activity (minutes/day, dichotomous), smoking (never, former, and current), alcohol intake (g/day, continuous), sleep duration (hours/day, continuous), treatment for hypertension (yes/no), depressive symptoms (continuous), and sodium intake (mg/day, continuous). Isocaloric substitution models- with a unit of change of 3% for all the macronutrients. When evaluating specific types of macronutrients, the rest of the macronutrients were included in the model. * $p < 0.05$.

Supplementary Table S3. Association between dietary fat substitution intake and arterial hypertension in the Health Workers Cohort Study 2004-2018.

	**Macronutrients intake**		Total sample,n=1451	Males, n=387	Females,n=1064
	**Increased**	**Decreased**	**OR(IC95%)**	**OR(IC95%)**	**OR(IC95%)**
Unadjusted	Saturated fat	LGI Carbohydrate	0.79 (0.70, 0.89)**	0.91 (0.70, 1.18)	0.74 (0.65, 0.85)**
Adjusted	Saturated fat	LGI Carbohydrate	0.88 (0.76, 1.00)	1.06 (0.79, 1.42)	0.83 (0.71, 0.97)*
Unadjusted	Monounsaturated fat	LGI Carbohydrate	1.11 (0.95, 1.30)	0.90 (0.64, 1.27)	1.16 (0.98, 1.39)
Adjusted	Monounsaturated fat	LGI Carbohydrate	1.29 (1.09, 1.53)**	1.04 (0.71, 1.52)	1.38 (1.13, 1.68)**
Unadjusted	Polyunsaturated fat	LGI Carbohydrate	0.71 (0.57, 0.88)**	0.86 (0.55, 1.34)	0.66 (0.51, 0.85)**
Adjusted	Polyunsaturated fat	LGI Carbohydrate	0.63 (0.50, 0.80)**	0.88 (0.54, 1.43)	0.57 (0.43, 0.76) **
Unadjusted	Saturated fat	HGI Carbohydrate	0.85 (0.75, 0.95)**	0.99 (0.77, 1.27)	0.81 (0.71, 0.93)**
Adjusted	Saturated fat	HGI Carbohydrate	0.87 (0.77, 1.00)	1.03 (0.78, 1.36)	0.83 (0.71, 0.96)*
Unadjusted	Monounsaturated fat	HGI Carbohydrate	1.17 (1.01, 1.36)*	0.98 (0.70, 1.38)	1.24 (1.04, 1.47)*
Adjusted	Monounsaturated fat	HGI Carbohydrate	1.29 (1.09, 1.52)**	1.03 (0.71, 1.50)	1.38 (1.13, 1.67)**
Unadjusted	Polyunsaturated fat	HGI Carbohydrate	0.76 (0.61, 0.95)*	0.94 (0.60, 1.47)	0.72 (0.55, 0.93)*
Adjusted	Polyunsaturated fat	HGI Carbohydrate	0.63 (0.49, 0.80)**	0.87(0.54, 1.42)	0.57 (0.43, 0.75)**

Generalized Estimating Equation (GEE) model. LGI: Low glycemic index carbohydrate- HGI: High glycemic index carbohydrate. Models referred to as “unadjusted” were adjusted for total energy intake (kcal/day continuous) and included all macronutrients except the one being replaced, as per the isocaloric substitution approach. The "adjusted" models included all the variables from the “unadjusted” models

(total energy intake and macronutrients except the one being replaced) as well as additional covariates: age (years, continuous), sex, physical activity (min/day, dichotomous), smoking (never, former, and current), alcohol intake (g/day, continuous), sleep time (hours/continuous), treatment for hypertension (yes/no), family history of hypertension, depressive symptoms (continuous), educational level and sodium intake (mg/continuous). Isocaloric substitution models- with a unit of change of 3% for all the macronutrients. When evaluating specific types of macronutrients, the rest of the macronutrients were included in the model. * $p < 0.05$- ** $p<0.01$.
